# Digital therapeutics using virtual reality‐based visual perceptual learning for visual field defects in stroke: A double‐blind randomized trial

**DOI:** 10.1002/brb3.3525

**Published:** 2024-05-21

**Authors:** Eun Namgung, Sun U. Kwon, Moon‐Ku Han, Gyeong‐Moon Kim, Hahn Young Kim, Kwang‐Yeol Park, Moonju Cho, Ha‐Gyun Choi, Hyun‐Wook Nah, Hyun Taek Lim, Dong‐Wha Kang

**Affiliations:** ^1^ Asan Institute for Life Sciences Asan Medical Center Seoul South Korea; ^2^ Department of Neurology, Asan Medical Center University of Ulsan College of Medicine Seoul South Korea; ^3^ Department of Neurology Seoul National University Bundang Hospital, Seoul National University College of Medicine Seongnam South Korea; ^4^ Department of Neurology, Samsung Medical Center Sungkyunkwan University School of Medicine Seoul South Korea; ^5^ Department of Neurology Konkuk University Medical Center, Konkuk University College of Medicine Seoul South Korea; ^6^ Department of Neurology Chung‐Ang University Hospital, Chung‐Ang University College of Medicine Seoul South Korea; ^7^ Nunaps Inc. Seoul South Korea; ^8^ Department of Neurology Chungnam National University Sejong Hospital, Chungnam National University College of Medicine Sejong South Korea; ^9^ Department of Ophthalmology, Asan Medical Center University of Ulsan College of Medicine Seoul South Korea

**Keywords:** chronic stroke, digital therapeutics, visual field defects, visual perceptual learning

## Abstract

**Introduction:**

Visual field defects (VFDs) represent a debilitating poststroke complication, characterized by unseen parts of the visual field. Visual perceptual learning (VPL), involving repetitive visual training in blind visual fields, may effectively restore visual field sensitivity in cortical blindness. This current multicenter, double‐blind, randomized, controlled clinical trial investigated the efficacy and safety of VPL‐based digital therapeutics (Nunap Vision [NV]) for treating poststroke VFDs.

**Methods:**

Stroke outpatients with VFDs (>6 months after stroke onset) were randomized into NV (defective field training) or Nunap Vision‐Control (NV‐C, central field training) groups. Both interventions provided visual perceptual training, consisting of orientation, rotation, and depth discrimination, through a virtual reality head‐mounted display device 5 days a week for 12 weeks. The two groups received VFD assessments using Humphrey visual field (HVF) tests at baseline and 12‐week follow‐up. The final analysis included those completed the study (NV, *n* = 40; NV‐C, *n* = 35). Efficacy measures included improved visual area (sensitivity ≥6 dB) and changes in the HVF scores during the 12‐week period.

**Results:**

With a high compliance rate, NV and NV‐C training improved the visual areas in the defective hemifield (>72 degrees^2^) and the whole field (>108 degrees^2^), which are clinically meaningful improvements despite no significant between‐group differences. According to within‐group analyses, mean total deviation scores in the defective hemifield improved after NV training (*p *= .03) but not after NV‐C training (*p *= .12).

**Conclusions:**

The current trial suggests that VPL‐based digital therapeutics may induce clinically meaningful visual improvements in patients with poststroke VFDs. Yet, between‐group differences in therapeutic efficacy were not found as NV‐C training exhibited unexpected improvement comparable to NV training, possibly due to learning transfer effects.

## Graphical abstract

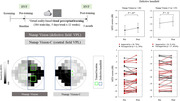

## 1 INTRODUCTION

Visual field defects (VFDs), characterized by unseen parts of the visual field, affect 20%–57% of people who experience a stroke (Pollock et al., 2019; Saionz et al., 2022). A VFD is usually unilateral, homonymous, and present as hemianopia (loss of one‐half of the visual field) or quadrantanopia (loss of one‐quarter of the visual field) depending on the lesion location (Pollock et al., 2019; Saionz et al., 2022). Most VFD recoveries occur within the first 3 months after injury, and spontaneous improvement is unlikely after 6 months without addressing the underlying disorders (Kim et al., 2019; Zhang et al., 2006). VFDs can hinder daily activities and diminish the overall quality of life (Gall et al., 2010; Papageorgiou et al., 2007). Despite the heavy burden of disability, there are scarce options for rehabilitation and recovery from VFDs (Sabel, 2006; Sagi, 2011).

Treatment and management approaches for people with VFDs can be described as compensation, substitution, and restitution (Kerkhoff, 2000; Pollock et al., 2019). Previous studies reported limited evidence of benefits from compensatory scanning and substitutive interventions (prisms); however, these interventions do not improve visual function in the defective field per se (Sabel, 2006; Sagi, 2011). Prism facilitates perception through refraction from a defect to an intact visual field; ocular training induces patients’ eyes to point to a defective visual field (de Haan et al., 2016; Rowe et al., 2017). Conversely, restitutive intervention, inducing visual field improvement by training, aims to regain visual perception in the defective field. Previous studies testing visual restitution training after a brain lesion indicated controversial results (Kasten et al., 1998; Reinhard et al., 2005).

Recently, an alternative approach was attempted by employing the principles of visual perceptual learning (VPL), which is defined as a long‐term improvement in performance on a visual task resulting from repeated visual training (Sagi, 2011; Sasaki et al., 2010). These studies revealed training performance improvement in the defective field and increased sensitivity in the perimetry (Cavanaugh & Huxlin, 2017; Huxlin et al., 2009). However, due to the small sample size and non‐randomized nature of the studies, visual restitutive interventions remain unsupported by extensive evidence (recommendation class IIb, level of evidence C) (Winstein et al., 2016). Accordingly, larger sized randomized trials are warranted to establish the efficacy of visual perceptual training in patients with stroke‐induced VFDs.

Ongoing debate surrounds the effectiveness of vision restoration methods, particularly in optimizing the VPL approach, including whether to prioritize detection or discrimination tasks, select types of stimuli, and target normal or blind areas (Lu & Dosher, 2022; Sagi, 2011; Saionz et al., 2022). It has been suggested that VPL with basic visual features (e.g., orientation and rotation) and location specificity involves early visual processing (e.g., primary visual cortex), whereas complex motion and depth discrimination tasks require visual decision‐making of higher order regions (e.g., MT, middle temporal area; LIP, lateral intraparietal area) (Gilbert et al., 2001; Sagi, 2011; Sasaki et al., 2010). Orientation‐motion discrimination tasks, sharpening tuning specificity of the primary visual cortex (V1), have mostly been applied in cortical blindness (Cavanaugh & Huxlin, 2017; Das et al., 2014; Huxlin et al., 2009). In previous studies, poststroke VFDs recovered after dual (peripheral orientation–central character) visual discrimination training, allowing central fixation (Lee et al., 2023; Namgung et al., 2024). Leveraging virtual reality (VR) can enhance the delivery, engagement, and performance of VPL (e.g., depth training) by offering immersive and controlled environments with customizable stimuli and interactive feedback (Godinez et al., 2021; Lin et al., 2022; Wilson & Soranzo, 2015).

Thus, based on previous findings (Lee et al., 2023; Namgung et al., 2024), we developed a new VR‐based visual perceptual training software (Nunap Vision [NV]), a digital therapeutics aimed at recovering VFDs poststroke. Using the VR device, which minimizes the potential effects of head movement, central and peripheral stimuli were simultaneously presented at a constant distance (Godinez et al., 2021; Lin et al., 2022; Wilson & Soranzo, 2015). Given the damaged visual pathway and occipital lobe in poststroke VFDs, orientation‐rotation‐depth discrimination training was chosen to facilitate task‐specific reweighting between basic visual representation and higher decision‐making stages (Dosher & Lu, 1998; Law & Gold, 2008). Considering the attention on defective fields (Ahissar, 2001; Schoups et al., 2001), we hypothesized and aimed to evaluate whether NV (defective field VPL with larger stimuli) can improve sensitivity in the defective field, measured by standard automated perimetry, in patients with chronic stroke‐induced VFDs compared to a matching Nunap Vision‐Control (NV‐C) (central field VPL with smaller stimuli).

## 2 METHODS

### 2.1 Participants and study design

**FIGURE 1:**
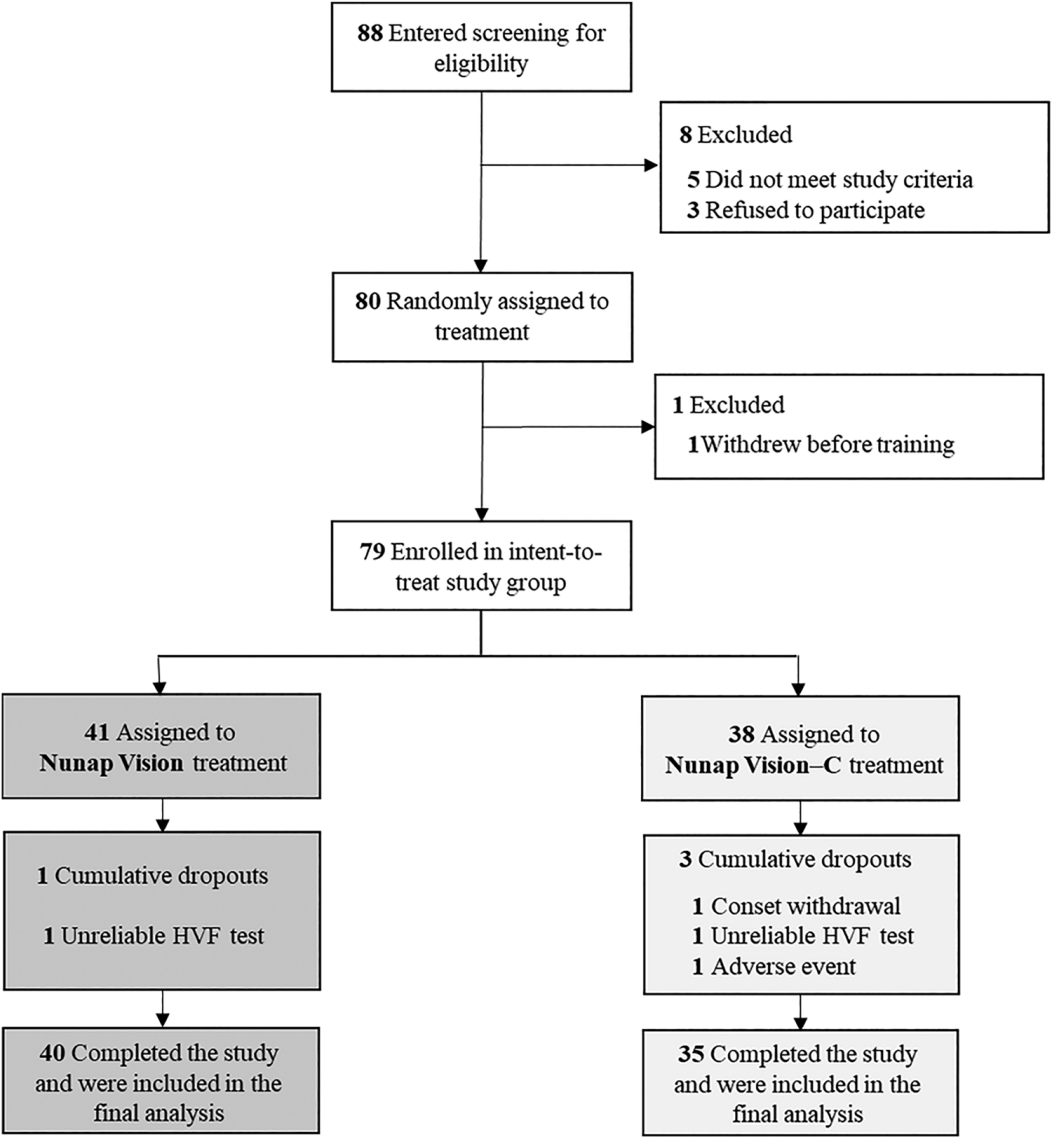
Flow diagram of study participants. Loss to follow‐up and withdrawal of consent was reasons for discontinuation. The dropout rates did not indicate significant differences between the two training groups. A total of 75 patients (94.9%) completed the trial. HVF, Humphrey visual field test; NV‐C, Nunap Vision‐Control.

This multicenter, double‐blind, randomized, controlled clinical trial (NCT04102605) was conducted from October 17, 2019 to May 31, 2021, in accordance with appropriate guidelines (Supplementary Material). A total of 88 outpatients with a VFD 6 months after stroke onset were recruited from 5 South Korean hospitals. A board‐certified ophthalmologist (H‐T.L) defined deficit visual fields according to the total deviation probability of <5% and VFD side and type based on the Humphrey visual field (HVF, 24‐2, SITA standard) test results (Acton et al., 2012; Barkana et al., 2021; Meditec, 2010). Written informed consent was obtained from the participants or their legally authorized representatives. As presented in Figure 1, eight patients were excluded after being screened for eligibility (*n* = 5, 5.68%) or withdrew (*n* = 3, 3.41%). One NV training group patient withdrew before receiving a training device. The detailed participant enrollment criteria and study design are described in Table S1.

### 2.2 Randomization and intervention

Within 1 month of signing the informed consent, the enrolled patients were randomized in a 1:1 ratio to NV (*n* = 41) or NV‐C (*n* = 38) using the randomization codes based on the computer‐generated permuted blocked procedure created by an independent contract research organization per clinical center.

**FIGURE 2:**
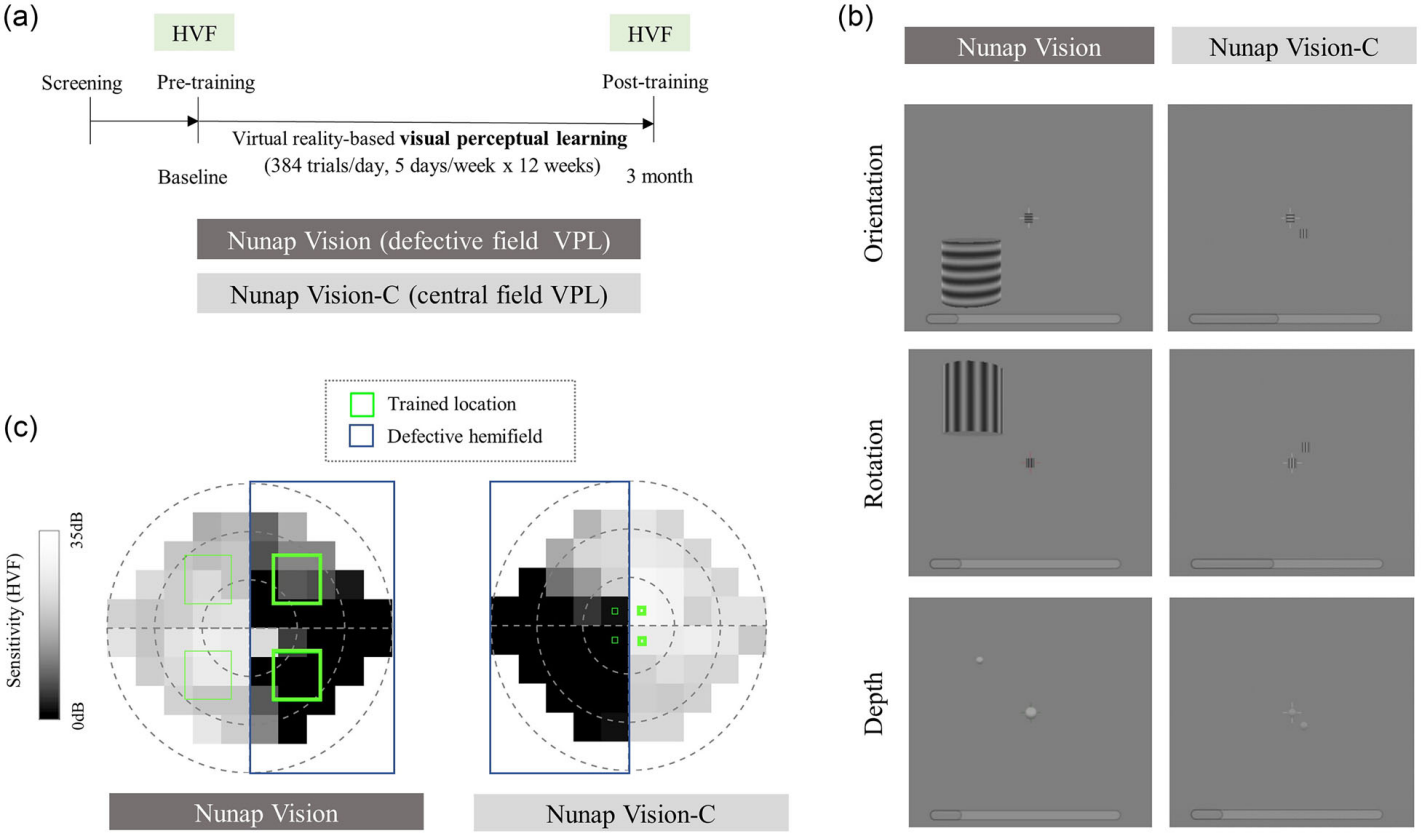
Study design and task procedures. (a) Schematic flow of study design. Patients were randomized into the Nunap Vision or Nunap Vision‐C training groups for 12 weeks. Visual field defects were assessed using Humphrey visual field tests at pre‐ and post‐training. (b) Schematic representation of the Nunap Vision (defective field training) and Nunap Vision‐C (central field training), which comprise orientation, rotation, and depth training using a virtual reality head‐mounted display. (c) Representation of peripheral stimuli presentation (trained location, green box) in the defective hemifield (blue box) in relation to the intact hemifield. Data for orientation discrimination training were visualized for two exemplary patients in the Nunap Vision and Nunap Vision‐C training groups. The darker visual points indicate lower sensitivity with more visual deficits measured using Humphrey visual field tests. The green box thicknesses indicate the frequency of the stimuli presentation in the four quadrants, with thicker boundaries indicating more frequent presentation. The Nunap Vision training visualized peripheral stimuli, Gabor cylinder (10 deg × 10 deg × 0.6 deg), in the defective hemifield 4.3 times more frequently than in the intact hemifield, whereas the Nunap Vision‐C training visualized it (0.6 deg × 0.6 deg × 0.6 deg) in in the intact hemifield 4.3 times more frequently than in the defective hemifield. deg, degrees; HVF, Humphrey visual field test; NV‐C, Nunap Vision‐Control; VPL, visual perceptual learning.

The intervention in both training groups (384 trials per day, 64 trials × 6 blocks per day, 5 days a week for 12 weeks, 60 sessions) was delivered at home using software providing VPL through a VR head‐mounted display (Oculus Go, Meta Inc., released May 2018; resolution per eye = 1280 × 1440 pixels, refresh rate = 60 Hz, field‐of‐view = 89 × 90 degrees) developed by Nunaps Inc. (Figure 2).

Both trainings provided the three types of visual perceptual training: orientation, rotation, and depth perception. Patients were asked to select any of the three types of training without specific rules. For each task, patients were presented with a middle‐gray blank with a beep sound (700 ms), visual stimuli located simultaneously in the central and peripheral quadrants (150 ms), and a response blank (3000 ms). The peripheral stimulus was presented randomly in each quadrant, and patients were required to press a button to indicate whether the central and peripheral stimuli had the same orientation, rotation, and depth (Figure 2). Auditory feedback for correct and incorrect responses was provided. Patients could choose practice sessions for any three training types before training began.

Gabor cylinders were used for orientation and rotation in the peripheral stimulus, whereas the depth training used white spheres. The NV‐C training group received the same instructions as the NV group. However, for the NV‐C training, the smaller peripheral stimuli were presented closer to the central field and 4.3 times more frequently in the intact hemifield compared to the NV training. The detailed parameters in the VPL protocol are described in Table S2. All data were initially stored locally and transferred to a server through a wireless network connection. Once the training was completed, research personnel could access the analysis.

### 2.3 Outcome measures

The primary outcome measure was the visual area measured using the HVF (24‐2, SITA‐standard), where sensitivity increased by 6 dB or more relative to baseline in the defective hemifield or whole field (Figure S1). Automated perimetry with the HVF is the gold standard in ophthalmology for accurately quantifying visual field sensitivity while controlling for fixation (Cavanaugh & Huxlin, 2017; Cavanaugh et al., 2021). We considered a visual sensitivity increase of 6 dB a significant change due to interventions, which roughly doubles the HVF test‐to‐test variability (Cavanaugh & Huxlin, 2017; Cavanaugh et al., 2021; Saionz et al., 2020), with a normal threshold of 30 dB for total deviation.

The secondary outcome measure was changes in the mean total deviation (MTD) scores relative to the baseline between and within the two training groups: MTD scores indicate differences in light detection results compared to age‐normative values.

We used the binocular‐integrated visual field method for the primary and secondary outcome measures: We constructed a single visual field map for each patient by selecting the highest sensitivity value from the visual fields of both eyes (Asaoka et al., 2011; Crabb et al., 2004).

### 2.4 Statistical analyses

Data normality was first tested using Shapiro–Wilk tests. Appropriate statistical analysis was performed according to the normality of the data.

The demographic and clinical characteristics of the study participants were compared between the two training groups using Mann‐Whitney *U* tests for continuous variables and Pearson's chi‐square tests or Fisher's exact tests for categorical variables.

Data analyses were conducted on an intent‐to‐treat basis, but the four patients (NV, *n* = 1; NV‐C, *n* = 3) who were dropped‐out without completing the 12‐week HVF test were excluded from the final statistical analysis. The primary and secondary outcome measures were performed on the patients who completed the trial (NV, *n* = 40; NV‐C, *n* = 35).

**FIGURE 3:**
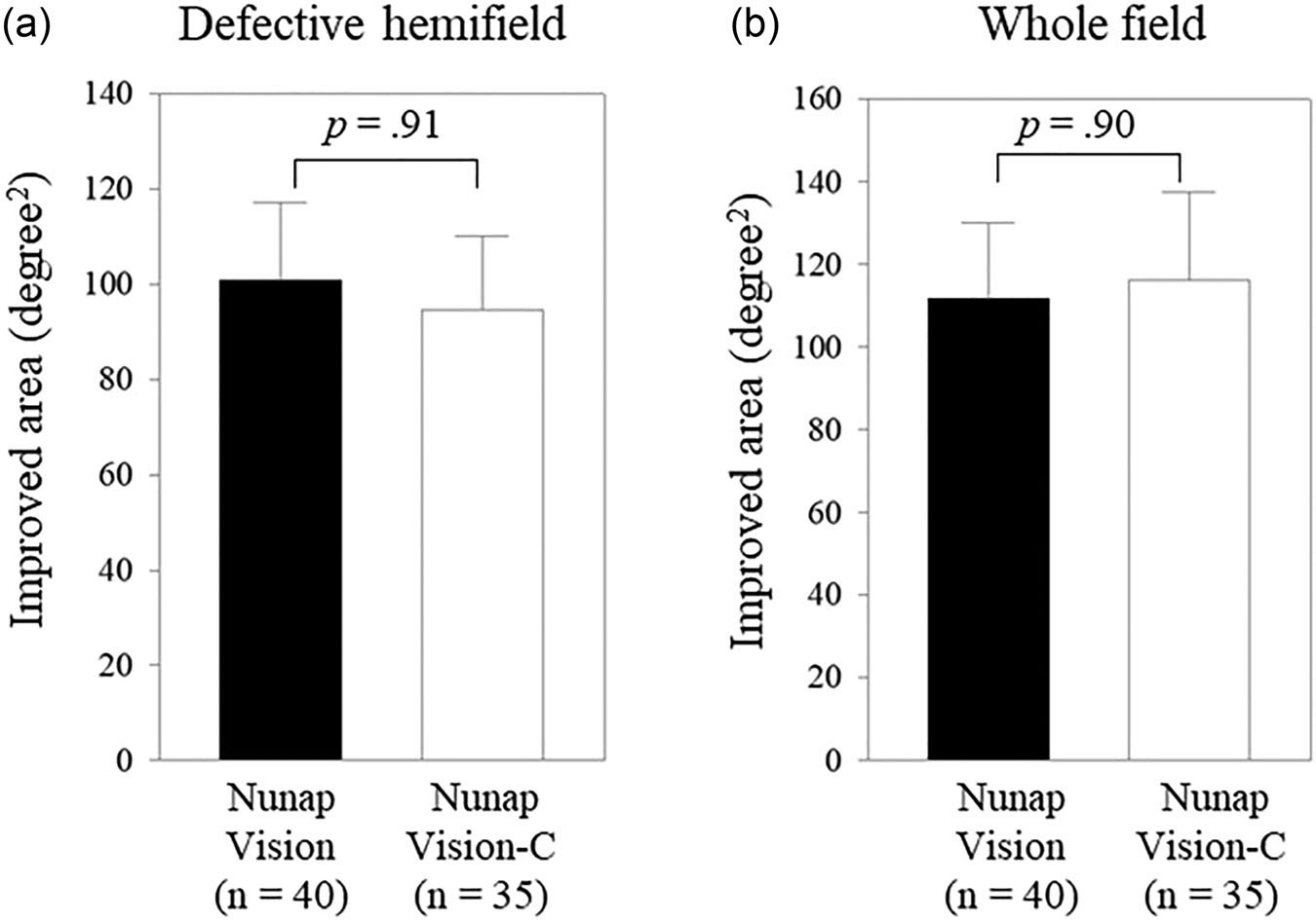
Primary outcome measures. After 12 weeks of training, the improved visual area (luminance detection sensitivity as measured by the Humphrey visual field test) in (a) the defective hemifield and (b) the whole field was compared between the Nunap Vision and Nunap Vision‐C training groups using Mann–Whitney *U* tests. The bar graphs indicate the mean values, and the error bars indicate the standard errors. NV‐C, Nunap Vision‐Control.

**FIGURE 4:**
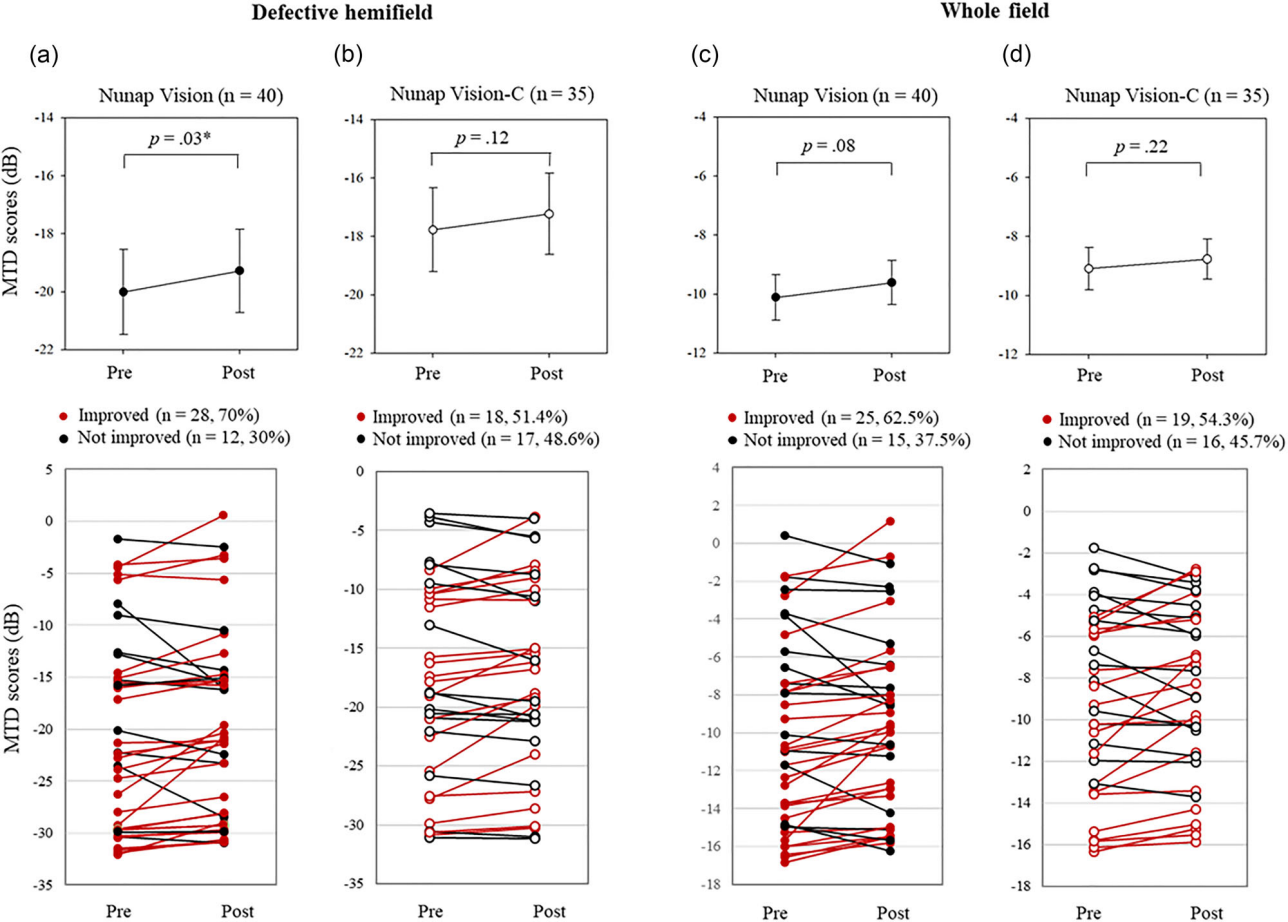
Secondary outcome measures: within‐group differences. The Wilcoxon signed‐rank test compared the mean total deviation scores in (a) the defective hemifield between pre‐ and post‐training within the Nunap Vision training group. Paired *t*‐tests were used for other within‐group analyses on (b) the defective hemifield within the Nunap Vision‐C training group and on (c, d) the whole field within both groups The mean and standard error averaged for each group are indicated as the circles and error bars on the top panels (Nunap Vision, black; Nunap Vision‐C, white). The redline indicates the patients who improved after the training, and the black line demonstrates those who did not improve after the training. MTD, mean total deviation; NV‐C, Nunap Vision‐Control.

**FIGURE 5:**
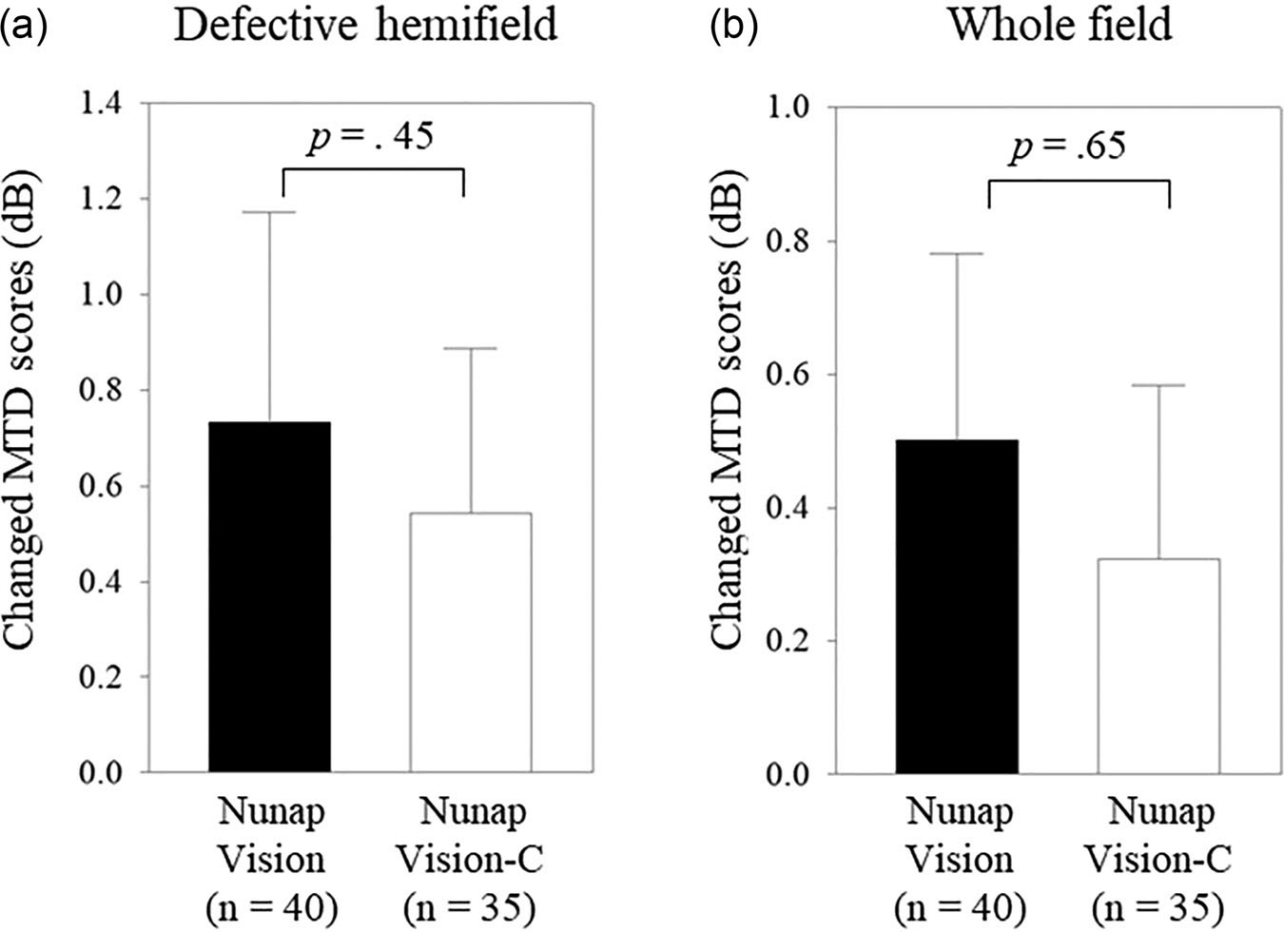
Secondary outcome measures: between‐group differences. Changed mean total deviation scores were compared between the Nunap Vision and Nunap Vision‐C groups using the Mann–Whitney *U* test for (a) the defective hemifield and the independent *t*‐test for (b) the whole field. The bar graphs indicate the mean values, and the error bars indicate the standard errors. MTD, mean total deviation; NV‐C, Nunap Vision‐Control.

The primary outcome measures were tested between the NV and NV‐C groups using Mann–Whitney *U* tests (Figure 3). For within‐group analysis on the secondary outcome measures, the pre‐ and post‐training MTD scores in the defective hemifield were compared within the NV training group using the Wilcoxon signed‐rank test (Figure 4a). Paired *t*‐tests were used for other within‐group analyses on the defective hemifield within the NV‐C group and the whole field within both groups (Figure 4b–d). Changed MTD scores in the defective hemifield were compared between the NV and NV‐C groups using the Mann–Whitney *U* test for the defective hemifield (Figure 5a) and the independent *t*‐test (Figure 5b) for the whole field.

## 3 RESULTS

### 3.1 Participant characteristics

The baseline demographic and clinical characteristics of the participants revealed no significant differences between the NV and NV‐C groups (Table 1). The reasons for discontinuation were as follows (Figure 1): unreliable HVF test results (NV, *n* = 1, 2.44%; NV‐C, *n* = 1, 2.63%), consent withdrawal (NV‐C, *n* = 1, 2.63%), and adverse event (NV‐C, *n* = 1, 2.63%). The dropout rates indicated no significant differences between the two groups (NV, *n* = 1, 2.44%; NV‐C, *n* = 3, 7.89%; *p *= .35); 75 patients (94.9%) completed the trial.

**TABLE 1:** Demographic and clinical characteristics of study participants.

	Total	Nunap Vision training	Nunap Vision‐C training	*p*
	(*n* = 75)	(*n* = 40)	(*n* = 35)	
Age (years)	52.01 ± 15.02	50.70 ± 14.29	53.51 ± 15.88	.32^a^
Male	44 (58.67)	26 (65.00)	18 (51.43)	.23^b^
Type of stroke				>.99^c^
Ischemic stroke	70 (93.3)	37 (92.50)	33 (94.29)	
Hemorrhagic stroke	5 (6.67)	3 (7.50)	2 (5.71)	
Hemifield of visual field defect				.85^b^
Left hemifield	42 (56.0)	22 (55.00)	20 (57.14)	
Right hemifield	33 (44.0)	18 (45.00)	15 (42.86)	
Type of visual field defect				
Homonymous hemianopia	40 (53.33)	26 (57.78)	19 (42.22)	.35^b^
Quadrantanopia	35 (46.67)	14 (46.67)	16 (53.33)	
Follow‐up from onset (years)	4.15 ± 3.93	3.90 ± 3.72	4.45 ± 4.20	.48^a^

*Note*: Data are presented as mean ± standard deviation and number (percentile).

^a^ Mann‐Whiteney *U* test

^b^ Pearson's chi‐square test.

^c^ Fisher's exact test.

### 3.2 Primary outcome measures

After 12 weeks of training, the mean (SD) improved area (luminance detection sensitivity ≥6 dB) in the defective hemifield was 100.8 (104.7) degrees^2^ in the NV group and 94.6 (92.0) degrees^2^ in the NV‐C group, indicating no significant between‐group differences (*p *= .91; Figure 3A). The improved area in the whole field was 111.6 (117.5) degrees^2^ in the NV group and 116.2 (126.0) degrees^2^ in the NV‐C group, indicating no significant between‐group differences (*p *= .90; Figure 3B). Both groups exhibited clinically significant improvements in the defective hemifield (>2 HVF points, 72 degrees^2^) and whole field (>3 HVF points, 108 degrees^2^).

### 3.3 Secondary outcome measures

#### 3.3.1 HVF changes in the defective hemifield and the whole field within the training groups

After the 12‐week training, the MTD scores significantly increased in the defective hemifield compared to the baseline within the NV group (pre, −20.0 ± 9.32; post, −19.3 ± 9.09; *p *= .03; Figure 4A). However, the MTD scores in the defective hemifield did not significantly change within the NV‐C group (pre, −17.8 ± 8.48; post, −17.2 ± 8.24; *p *= .12; Figure 4B); 28 participants in the NV group demonstrated training‐induced‐improved MTD scores in the defective hemifield (70%; Figure 4A), with 18 (51.4%; Figure 4B) in the NV‐C group.

After the 12‐week training, the MTD scores did not significantly change in the whole field compared to the baseline within the NV group (pre, −10.1 ± 4.90; post, −9.61 ± 4.74; *p *= .08; Figure 4C). Moreover, the MTD scores in the whole field did not significantly change within the NV‐C group (pre, −9.09 ± 4.28; post, −8.77 ± 4.05; *p *= .22; Figure 4D); 25 participants in the NV group demonstrated training‐induced‐improved MTD scores in the defective hemifield (62.5%; Figure 4C), with 19 (54.3%; Figure 4D) in the NV‐C group.

#### 3.3.2 HVF changed scores between the training groups

The NV and NV‐C groups exhibited no significant differences in the training‐induced changes in the MTD scores in the defective hemifield (NV = .73 ± 2.78, NV‐C = .54 ± 2.04; *p *= .45; Figure 5A) and whole visual field (NV = .50 ± 1.77, NV‐C = .32 ± 1.54; *p *= .65; Figure 5B).

#### 3.3.3 VPL performance and safety measures

During the 12‐week training, VPL showed overall improvement in orientation (*p* < .001), rotation (*p* < .001), and depth (*p* = .03) dimensions despite fewer completions of the depth training (Table S3). The magnitude of overall improvement in the orientation training was greater for the NV group than for the NV‐C group (*p* < .001, Table S4). During the 12‐week training, the increased correct responses in the orientation training were positively associated with improved defective hemifield only in the NV group (*p* = .046). All reported adverse events are presented in Table S5.

## 4 DISCUSSION

This study aimed to provide evidence for the safety and efficacy of newly developed digital therapeutics based on visual perceptual training for stroke‐induced VFDs, which required proven training strategies. NV (targeting defective visual field) and NV‐C (targeting central visual field) training demonstrated high compliance rates. The MTD scores in the defective field improved only after the NV training compared to pre‐training and not after the NV‐C training. Unexpectedly, the NV‐C training led to clinically significant improved areas (sensitivity ≥6 dB) comparable to the NV training, potentially due to learning transfer effects.

Given the chronicity of poststroke VFDs in this study and the significant worsening, defined as <3 HVF points in glaucoma (Leske et al., 1999), the NV and NV‐C groups exhibited clinically meaningful improvement in the whole field (>3 HVF points, 108 degrees^2^). MTD scores in the defective hemifield improved only after the NV training, and increased correct responses in the orientation training were positively associated with the improved defective hemifield only in the NV group. Therefore, frequent visualization of Gabor for orientation discrimination tasks within defective visual fields (NV training) may enhance tuning specificity in lesioned V1 cells, resulting in targeted improvement within the defective hemifield (Sasaki et al., 2010; Schoups et al., 2001). The greater improvement in the orientation training in the NV group further supports this. Despite variations in methodology between research studies, the magnitude of visual improvement after the NV training was superior to prior control conditions (no‐training) and comparable to previous defective field training (Bergsma & Van der Wildt, 2010; Cavanaugh & Huxlin, 2017; Cavanaugh et al., 2021; Sahraie et al., 2006). The VFD improvement within the defective area resembled that in the defective hemifield, implying that VPL and VFD improvements extend across visual fields, encompassing defective areas and boundaries between normal and defective areas (Cavanaugh et al., 2015; Das et al., 2014; Huxlin et al., 2009; Sabel & Kasten, 2000).

As a noninvasive VR‐based VPL software, NV was designed to target the neuroplasticity of the V1 through interactive bottom‐up and top‐down mechanisms (Sasaki et al., 2010; Urbanski et al., 2014): VPL results from task‐specific changes in the strength of neural connections between low‐level visual representation and higher decision‐making stages (Dosher & Lu, 1998; Law & Gold, 2008). First, the orientation discrimination task of Gabor with spatial frequency may reshape early visual processing, including the tuning properties of the V1 retinotopically corresponding to the trained stimulus location (Sasaki et al., 2010; Schoups et al., 2001). Neural representations of visual stimuli may be enhanced through synaptic strengthening and dendritic remodeling (Gilbert et al., 2001; Karmarkar & Dan, 2006). Second, VPL affects connectivity between the visual cortex and higher regions involved in decision‐making, including MT and LIP, through top‐down cognitive modulation (Dosher & Lu, 1998; Law & Gold, 2008). After NV training, the damaged V1 and higher visual regions (MT and LIP) involved in depth discrimination and decision‐making may be effectively stimulated, inducing visual restoration (Dosher & Lu, 1998; Law & Gold, 2008). Previously, VFD showed improvement by modifying the connectivity of the lesioned visual cortex with contralateral visual cortex and temporal regions (Kang et al., 2018; Kim et al., 2015; Namgung et al., 2024).

This study is the largest clinical trial using a multicenter, double‐blind, randomized, controlled design capable of minimizing potential biases. We included VFD patients who had a stroke more than 6 months previously to minimize the potential effects of spontaneous VFD recovery, mostly occurring within the first 3 months. Sampling bias was minimized using strict randomization and recruiting patients from multiple medical centers. Being blinded to treatments was maintained using identical instructions and user hardware and software interfaces on both training devices. Furthermore, an alternative (NV‐C) to sham training was used as the control, resulting in high training compliance and lower dropouts. Moreover, comparing the outcomes of the two training approaches, which target the defective and central fields, could provide valuable insights into optimizing VPL protocols, including the location and visual stimuli sizes. From an ethical standpoint, this approach aligns with the aim of the trial, which was to develop safe and effective VPL‐based training for stroke‐related VFDs. Using the VR head‐mounted display at home maximized accurate localization of the briefly presented visual stimuli with a constant viewing distance, potentially offering highly controlled and realistic training environments.

Contrary to our expectation, improved visual area and training‐induced HVF changes indicated no significant differences between the NV and NV‐C groups; this might be because we compared it against the central field training instead of the untrained controls or sham training. The NV‐C training, expected to induce minimal changes as the control, improved the visual area as a less effective intervention than a true placebo (Cavanaugh et al., 2021; Elshout et al., 2016). Variations in sample size, HVF test types, and VPL methodology may potentially explain the differences in the improved visual area reported across studies (Cavanaugh & Huxlin, 2017; Lee et al., 2023). Alternatively, learning transfer effects, occurring in an easier and repetitive task, might have occurred during NV‐C training (Bergsma & Van der Wildt, 2010; Cavanaugh & Huxlin, 2017; Cavanaugh et al., 2019). Furthermore, smaller peripheral stimuli were visualized within 5 degrees of the central visual field and more often in the intact hemifield with an easier level in the NV‐C than the NV. In the NV‐C training, reward‐based reinforcement may facilitate implicit and task‐irrelevant VPL by enhancing diffusive signals across all stimuli, leading to visual recovery unspecific to the defective hemifield (Carrasco et al., 2008; Gutnisky et al., 2009). Conversely, the NV training may lead to task‐relevant VPL, particularly in defective fields: sustained attention to the defective hemifield may enhance task‐relevant signals directed to a specific spatial location in the brain, while inhibiting task‐irrelevant signals (Ahissar, 2001; Schoups et al., 2001).

NV offers a noninvasive treatment with potential long‐term effects on visual functions but requires extensive time and resources (Lin et al., 2022; Lu & Dosher, 2022; Wilson & Soranzo, 2015). Other treatment options, including visual prosthetic devices and noninvasive neuromodulation, may provide immediate restoration by specifically targeting damaged neural pathways, although they have high costs and limited generalization (Lu & Dosher, 2022; Sagi, 2011; Saionz et al., 2022). Adjunctive pharmacology (e.g., selective serotonin reuptake inhibitors) and noninvasive neuromodulation (e.g., transcranial direct current stimulation), which have been applied alone or alongside VPL, lacked consensus on the safety and efficacy across different recovery stages and optimal usage for chronic cortical blindness (Alber et al., 2017; Cavanaugh & Huxlin, 2017; Dennis et al., 2019; Saionz et al., 2022).

Future directions for practical applications of VPL in cortical blindness include (1) optimizing VPL integration into existing rehabilitation programs with maximized therapeutic benefits by extending the poststroke‐sensitive period and facilitating neuroplasticity; (2) conducting longitudinal studies to track real‐world visual functions over time and elucidating mechanisms underlying skill retention, informing the development of improved training protocols; (3) exploring cross‐modal training paradigms by combining visual stimuli with auditory or tactile cues to enhance neural plasticity and sensory processing efficiency; and (4) identifying biomarkers associated with VPL response, spanning from biological to neuroimaging markers, to facilitate the development of personalized intervention strategies through predictive modeling (Lu & Dosher, 2022; Sagi, 2011; Saionz et al., 2022).

This study has some limitations that need to be considered. First, training compliance could not be objectively monitored through real‐time feedback because cyber security regulations for digital therapeutics were not established during this trial. However, training compliance, a core element governing the effectiveness of digital therapeutics, was monitored throughout the trial, and feedback was provided to participants who did not fulfill the guidance. Additionally, hands‐on professional assistance may be limited and inconsistent in our clinical study testing VPL software using at‐home VR devices. Moreover, clinical history, including comorbidity and medication history, could not be controlled in our study. Despite excluding participants with fixation loss, false positive, and false negative ≥20%, potential eye movement could not be measured without eye trackers. Although we reliably measured luminance detection using the standard HVF, other visual performances should be assessed, such as contrast sensitivity, which is not limited to luminance detection. Future larger studies that provide eye tracking, objective measures of training and compliance, visual measures other than luminance detection, and sham training as the control are warranted for the generalizability of the study findings.

Notably, this multicenter, double‐blind, randomized controlled clinical trial is the largest to evaluate the safety and efficacy of VR‐based visual perceptual training for stroke‐related VFDs. Along with a high compliance rate, NV training demonstrated significantly improved stroke‐related VFDs, despite NV‐C training unexpectedly improving sensitivity comparable to NV; this could be due to learning transfer effects. The current findings may provide insights into developing a novel visual restitutive strategy based on VPL.

## AUTHOR CONTRIBUTIONS

Dong‐Wha Kang participated in the study design, had full access to all the data in the study, and was responsible for the integrity of the data and the accuracy of the data analysis. Eun Namgung, Sun U. Kwon, Hyun‐Wook Nah, and Dong‐Wha Kang drafted the manuscript. Eun Namgung, Moonju Cho, and Ha‐Gyun Choi acquired, analyzed, and interpreted the data; Moon‐Ku Han, Gyeong‐Moon Kim, Hahn Young Kim, and Kwang‐Yeol Park provided administrative, technical, or material support. Eun Namgung, and Dong‐Wha Kang obtained the funding. Sun U. Kwon, Hyun‐Wook Nah, and Hyun Taek Lim supervised the study. All authors reviewed and approved the manuscript.

## CONFLICT OF INTEREST STATEMENT

The authors declare no conflicts of interests.

## FUNDING INFORMATION

Korea Health Industry Development Institute (KHIDI) funded by the Ministry of Health & Welfare (HR18C0016); National Research Foundation of Korea (NRF) funded by the Korean government (MSIT) (2022R1F1A1060778).

### PEER REVIEW

The peer review history for this article is available at https://publons.com/publon/10.1002/brb3.3525.

## PATIENT CONSENT STATEMENT

Written informed consent was obtained from the participants or their legally authorized representatives.

## CLINICAL TRIAL REGISTRATION

ClinicalTrials.gov, NCT04102605. Registered September 25, 2019, https://clinicaltrials.gov/ct2/show/NCT04102605.

## Supporting information

Table S1 Schedule of enrollment, interventions, and assessments.
Table S2 Specification and parameters of visual perceptual learning.
Table S3 Performance changes in visual perceptual learning for 12 weeks.
Table S4 Between‐group differences in visual perceptual learning performance changes.
Table S5 Adverse events (safety analysis set).
Figure S1 Study outcome measures.

## Data Availability

Following publication, deidentified data can be shared upon reasonable request and a methodologically sound proposal to the corresponding author.
