# Supplementary material for: Digital therapeutics using virtual reality‐based visual perceptual learning for visual field defects in stroke: A double‐blind randomized trial
Source: Brain Behav. 2024 May 21;14(5):e3525. doi: 10.1002/brb3.3525 (PMC11109502; doi:10.1002/brb3.3525)
Supplement: Supplementary file 1 — Table S1 Schedule of enrollment, interventions, and assessments. Table S2 Specification and parameters of visual perceptual learning. Table S3 Performance changes in visual perceptual learning for 12 weeks. Table S4 Between‐group differences in visual perceptual learning performance changes. Table S5 Adverse events (safety analysis set). Figure S1 Study outcome measures. [file BRB3-14-e3525-s001.docx]

**SUPPLEMENTARY MATERIAL**

Digital Therapeutics Using Virtual Reality-based Visual Perceptual Learning for Visual Field Defects in Stroke: A Double-blind Randomized Trial

SUPPLEMENTARY METHODS

SUPPLEMENTARY RESULTS

SUPPLEMENTARY REFERENCES

Table S1. Schedule of enrollment, interventions, and assessments

Table S2. Specification and parameters of visual perceptual learning

Table S3. Performance changes in visual perceptual learning for 12 weeks

Table S4. Between-group differences in visual perceptual learning performance changes

Table S5. Adverse events (safety analysis set)

Figure S1. Study outcome measures

**SUPPLEMENTARY METHODS**

**Participants and study design**

This multicenter, double-blind, randomized, controlled clinical trial was initiated following the Ministry of Food and Drug Safety (NCT04102605, 25/09/2019) and approved by the Institutional Review Boards at Asan Medical Center (IRB No. 2019-1186), Seoul National University Bundang Hospital (E-1908/558-001), Samsung Medical Center (SMC2019-06-088-001), Konkuk University Medical Center (KUMC2019-12-021-001), and Chung-Ang University Hospital (1923-001-401). The study was conducted from October 17, 2019, to May 31, 2021, in accordance with the Declaration of Helsinki, complying with the Standard Protocol Items: Recommendation for Interventional Trials statement and Consolidated Standards of Reporting Trials criteria.

The enrolled participants were patients aged 19–80 years who have experienced stroke-induced visual field defects (VFDs) for more than 6 months and have visual pathway damage confirmed by brain imaging, a Mini-Mental Status Examination score ≥ 24, and a visual acuity ≥ 20/40. The exclusion criteria included an unreliable Humphrey visual field (HVF, 24-2, SITA standard) test (any of fixation loss, false positive, false negative ≥ 20%), complete hemianopia with a sensitivity of the whole hemifield ≤ 3 dB, bilateral VFDs, hemispatial neglect, comorbid neurological disorders or ophthalmologic disorders that may interfere with the trial, an inability to discontinue psychostimulants, candidacy for carotid endarterectomy or stenting, a history of ophthalmologic surgery within the preceding three months, and current pregnancy or breast-feeding.

There were four clinical visits in the trial (Table S1). At the first visit, informed consent was provided after eligibility screening; the demographic and clinical characteristics were assessed. Within 28 days of the screening, the HVF test was performed as the baseline visit for pre-training assessment, and the investigational device was delivered. Then, safety measures and training compliance were assessed 6 weeks after the baseline (± 14 days). Patients were re-educated on the usage of the device if needed. HVF was performed as the post-training assessment 12 weeks after the baseline (± 7 days), and the investigational device was returned.

For sample size estimation, the null hypothesis is that the mean area of HVF where sensitivity increases by ≥ 6 dB relative to baseline will not significantly differ between the two groups. A sample size of 50 patients in each trial group is required to determine the primary outcome based on an alpha level of 0.05, a beta level of 0.20, a standard deviation of 109.7 degrees^2^, and the minimum mean difference of 61.9 degrees^2^ between the visual perceptual learning (VPL) training and no-training groups. The mean and standard deviation was based on our proof of concept study, which compared a dual peripheral orientation-central letter discrimination task with the no-training on poststroke VFDs using HVF test (30-2 SITA standard).

**Intervention: visual perceptual learning protocol**

The instructions for VPL were visualized on the virtual reality head-mounted display: "Press the thumb button if the central and peripheral stimuli have the same orientation, rotation, and depth. Press the index finger button if the central and peripheral stimuli have a different orientation, rotation, and depth".

For the Nunap Vision Control (NV-C) training, the peripheral stimuli were smaller in size for cylinders (NV, 10 degrees; NV-C, 0.6 degrees in the height, width, and diameter for cylinders), presented closer to the central field (NV, ± 10 degrees^2^; NV-C, ± 5 degrees^2^ of the central visual field), and were more frequently in the intact hemifield (NV, defective hemifield: intact hemifield = 4.3:1; NV-C, defective hemifield: intact hemifield = 1:4.3) compared to the Nunap Vision (NV) training.

For orientation and rotation, the spatial frequency of the Gabor cylinder was designed to be 2.5 cycles/degree at the center and 0.5 cycles/degree in the periphery. The speed for rotation was 25 degrees per second. Depth training was performed using a white sphere with a diameter of 1.2 degrees for both the NV and NV-C training groups. Depth training varied by ± 10 centimeters from the base point, which is 40 centimeters. The colors for the stimuli were as follows: cylinders, RGB (255,255,255); spheres, central RGB (88,88,88), and peripheral RGB (180,180,180) (Table S2).

The difficulty level was adjusted by varying the contrast-to-background intensity of the gradient pattern across 15 levels exponentially to account for individual variances in VPL performance. This adjustment followed a 1-up/3-down staircase procedure. With a decreasing factor of 0.7, the contrast level decreased to 70% after three consecutive correct responses from the initial 100% and increased to 100% after an incorrect response.

**Visual perceptual learning and visual field defect improvement**

The VPL performance was computed using the difficulty level, correct responses, and overall performance in the total participants (n = 75), NV training group (n = 40), and NV-C training group (NV-C, n = 35) for the first week and 12 weeks after. The first week indicates the completion of the first block (64th trial), and the 12 weeks indicate the completion of the last sixth block (64th trial). Difficulty levels (1–15) were calculated by averaging those in the four quadrants, with the higher numbers indicating enhanced difficulty and contrast levels. Correct responses (%) were calculated within one block of 64 trials. Overall performance was calculated by multiplying the correct responses with the difficulty level divided by 15.

A linear mixed-effects model was used to examine the changes in the VPL performance (difficulty level, correct responses, overall performance) between 1 week and 12 weeks within the total participants (n = 75), NV training group (n = 40), and NV-C training group (n = 35), respectively (Table S3). Mann–Whitney U tests compared the 12-week changes in VPL performance between the NV and NV-C training groups (Table S4). Regression analyses using age and sex as covariates were performed to investigate the relationships between VPL performance (difficulty level, correct responses, overall performance) and VFD improvement (improved area in the defective hemifield and whole field, changed MTD scores in the defective hemifield and whole field). Regression analyses were repeated within the total participants (n = 75), NV training group (n = 40), and NV-C training group (n = 35).

**Outcome measures within the defective and intact visual area**

The individual defective visual area was computed by combining abnormal visual points from both eyes with a total deviation probability of < 5%, occurring in less than 5% of healthy individuals, according to the HVF test results (24-2, SITA standard) (Acton, Smith, Greenberg, & Greenstein, 2012; Barkana et al., 2021; Meditec, 2010). The intact visual area was calculated by excluding the defective visual fields from the whole field.

Within the individual defective and intact areas, improved area (luminance detection sensitivity ≥ 6 dB) and changed MTD scores were calculated for the 12-week training period and compared between the NV (n = 39) and NV-C (n = 35) training groups using Mann–Whitney U tests. Wilcoxon signed-rank tests were used to compare MTD scores in the defective area between pre- and post-training, within the NV (n = 39) and NV-C (n = 35) training groups, respectively.

**Safety and tolerability outcome measures**

As tolerability measures, training compliance was monitored by a medical device usage log written by patients for each training usage and reviewed by an investigator during the interview at each visit, 6 weeks and 12 weeks after the allocation. Compliance was calculated by the ratio of actual medical device usage divided by recommended usage of medical devices (60, 5 days a week for 12 weeks).

As for safety measures, adverse events were evaluated by clinical interview, vital signs, and physical examination at each of the four visits (Table S5).

The safety and tolerability measures were analyzed in all patients randomly assigned to a training group with at least one efficacy assessment after randomization (NV, n = 41; NV-C, n = 38). Proportions of adverse events were compared between the two groups using Fisher's exact tests. Tolerability was compared between the two groups using a Mann–Whitney *U* test.

**SUPPLEMENTARY RESULTS**

**Visual perceptual learning and visual field defect improvement**

Overall performance of VPL, multiplied by the relative difficulty level and correct responses, significantly improved for the 12 weeks of training in the orientation (*p* < .001), rotation (*p* < .001), and depth (*p* = .03) dimensions. The difficulty level significantly increased for the 12-week training in the orientation (*p* < .001) and rotation (*p* < .001) dimensions but not in depth (*p* = .07). The correct responses showed significant improvement for the 12 weeks in the orientation (*p* < .001), rotation (*p* < .001), and depth (*p* = .001) dimensions (Table S3).

The 12-week improvement in the overall learning performance of the orientation training was significantly higher in the NV training group than in the NV-C training group (*p* < .001). However, the 12-week overall performance changes in rotation (*p* = .67) and depth (*p* = .85) training showed no significant differences between the NV and NV-C training groups. The NV training group showed greater improvement in the difficulty level for the 12-week training regarding the orientation (*p* < .001), but not for the rotation (*p* = .64) and depth (*p* = .80), than the NV-C training group. The 12-week increase in the correct responses was higher for the rotation (*p* < .001) but not for the orientation (*p* = .90) and depth (*p* = .70) in the NV-C training group than in the NV training group (Table S4).

During the 12-week training period, the increased correct response to the orientation training was positively associated with the improved defective hemifield in the NV training group with age and sex as covariates (*β* = .350, *p* = .046). No significance was observed in the NV-C training group (*β* = .025, *p* = .887). The 12-week changes in other VPL performance metrics were not associated with the improved area in the whole field and defective hemifield and changed MTD scores in the whole field and defective hemifield.

**Outcome measures within the defective visual area**

After 12 weeks of training, the mean (standard deviation) improved area (luminance detection sensitivity ≥ 6 dB) in the defective visual area was 107 (111) degrees^2^ in the NV training group and 98.7 (102) degrees^2^ in the NV-C training group, indicating no significant between-group differences (*p* = .66).

The NV and NV-C training groups exhibited no significant differences in the changed MTD scores within the defective area (NV = 1.20 ± 3.93, NV-C = 1.03 ± 2.52; *p* = .70).

After the 12-week training, the MTD scores in the defective visual area significantly improved compared to the baseline within the NV training group (pre, -23.9 ± 6.70; post, -22.7 ± 7.84; *p* = .011) and NV-C training group (pre, -22.5 ± 6.39; post, -21.4 ± 6.95; *p* = .018).

**Outcome measures within the intact visual area**

After 12 weeks of training, the mean (standard deviation) improved area (luminance detection sensitivity ≥ 6 dB) in the intact visual area was 4.62 (14.7) degrees^2^ in the NV training group and 15.4 (44.8) degrees^2^ in the NV-C training group, indicating no significant between-group differences (*p* = .70).

The NV and NV-C training groups exhibited no significant differences in the changed MTD scores within the intact area (NV = -0.10 ± 1.62, NV-C = -0.18 ± 1.55; *p* = .34).

After the 12-week training, the MTD scores in the intact visual area did not significantly change compared to the baseline within the NV training group (pre, -0.03 ± 1.41; post, -0.13 ± 1.27; *p* = .93;) and NV-C training group (pre, -0.37 ± 1.25; post, -0.55 ± 1.44; *p* = .18).

**Compliance and safety**

The NV and NV-C training group patients exhibited high compliance (NV, 94.4 ± 17.9 %; NV-C, 98.5 ± 22.0 %; *p* =.28). All reported adverse events are presented in Table S5. The overall frequencies of the adverse events in the two training groups revealed no significant differences (NV, n = 6, 14.6 %; NV-C, n = 5, 13.2 %; *p* > 0.99).

The adverse events related to the medical device were nausea (n = 1, 1.27 %), vomiting (n = 1, 1.27 %), dizziness (n = 3, 3.80 %), and headache (n = 6, 7.59 %). In addition, two patients in the NV-C training group revealed serious adverse events and fully recovered after receiving proper medical training through admission to hospitals, carotid artery stenosis (n = 1, 2.63 %), and cerebral hemorrhage (n = 1, dropped out, 2.63 %). Alternatively, there were no serious or unanticipated adverse device effects.

**SUPPLEMENTARY REFERENCES**

Acton, J. H., Smith, R. T., Greenberg, J. P., & Greenstein, V. C. (2012). Comparison between MP-1 and Humphrey visual field defects in glaucoma and retinitis pigmentosa. *Optometry and Vision Science, 89*(7), 1050-1058.

Barkana, Y., Leshno, A., Stern, O., Singer, R., Russ, H., Oddone, F., . . . Garway-Heath, D. F. (2021). Visual Field Endpoints Based on Subgroups of Points May Be Useful in Glaucoma Clinical Trials: A Study With the Humphrey Field Analyzer and Compass Perimeter. *Journal of Glaucoma, 30*(8), 661-665.

Lee, E. J., Kim, D., Kim, Y. H., Namgung, E., Lee, J. H., Sasaki, Y., . . . Kang, D. W. (2023). Digital Therapeutics With Visual Discrimination Training for Cortical Blindness in Patients With Chronic Stroke. *J Stroke, 25*(3), 409-412. doi:10.5853/jos.2023.00276

Meditec, C. Z. (2010). Humphrey Field Analyzer II-i Series, User Manual. *Dublin, Ireland: Carl Zeiss Meditec Inc*.

**Table S1. Schedule of enrollment, interventions, and assessments.**

|  | **Study period** | | | |
| --- | --- | --- | --- | --- |
|  | **Enrollment** | **Allocation** | **Post-allocation** | **Close-out** |
| Time point | *-4 w* | 0 | *6 w ± 14d* | *12w ± 7d* |
| **Enrollment** |  |  |  |  |
| Eligibility screen | X |  |  |  |
| Informed consent | X |  |  |  |
| Allocation |  | X |  |  |
| **Interventions** |  |  |  |  |
| Nunap Vision training |  | X | X |  |
| Nunap Vision-C training |  | X | X |  |
| Compliance check |  |  | X | X |
| **Assessments** |  |  |  |  |
| Demographics,  past medical history | X |  |  |  |
| Vital signs,  physical examination | X | X | X | X |
| Humphrey visual fields tests |  | X |  | X |

Abbreviations: d, days; w, weeks; Nunap Vision-C, Nunap Vision-Control.

**Table S2. Specification and parameters of visual perceptual learning.**

| **Parameters** | **Nunap Vision training** | **Nunap Vision-Control training** |
| --- | --- | --- |
| Central stimulus: types (dimensions^1^) | - Orientation: cylinder (height = 0.6 deg, width = 0.6 deg, diameter = 0.6 deg)  - Rotation: cylinder (height = 0.6 deg, width = 0.6 deg, diameter = 0.6 deg)  - Depth: sphere (radius = 0.6 deg) | |
| Peripheral stimulus: types (dimensions^1^) | - Orientation: cylinder (height = 10 deg, width = 10 deg, diameter = 10 deg)  - Rotation: cylinder (height = 10 deg, width = 10 deg, diameter = 10 deg)  - Depth: sphere (radius = 0.6 deg) | - Orientation: cylinder (height = 0.6 deg, width = 0.6 deg, diameter = 0.6 deg)  - Rotation: cylinder (height = 0.6 deg, width = 0.6 deg, diameter = 0.6 deg)  - Depth: sphere (radius = 0.6 deg) |
| Peripheral stimulus: location | Eccentricity and (X,Y) cartesian coordinates of the stimulus center relative to fixation | |
|  | ± 10 degrees^2^ of the central visual field  (10,10) (-10,10)(-10,-10)(10,-10) | ± 5 degrees^2^ of the central visual field  (5,5) (-5,5)(-5,-5)(5,-5) |
| Peripheral stimulus: presentation | Presentation in defective hemifield to that in intact hemifield | |
|  | Defective hemifield: intact hemifield = 4.3:1 | Defective hemifield: intact hemifield = 1:4.3 |
| Screen luminance | - Screen (Default setting for Oculus Go): resolution per eye = 1280 x 1440 pixels, refresh rate = 60 Hz  - Window covering stimuli: R = 128, G = 180, B = 128  - Cylinder (orientation, rotation): R = 255, G = 255, B = 255  - Sphere (depth); periphery, R = 88, G = 88, B = 88; core, R = 180, G = 180, B = 180 | |
| Stimulus contrast | 15 levels (1-up/3-down staircase procedure)  - Adjusted by decreasing factor of 0.7 from the initial 100% | |
| Speed of rotation | 25 degrees per second | |
| Spatial frequency  (orientation, rotation) | Sinusoidal waveform  - Central stimuli: 2.5 cycles per degree  - Peripheral stimuli: 0.5 cycles per degree  (stimuli for orientation and rotation, not for depth training) | |
| Positioning in depth | 40 ± 10 cm  (±10 centimeters from the base point, which is 40 centimeters) | |

^1^ Apart from 40 cm distance.

Abbreviations: deg, degrees.

**Table S3. Performance changes in visual perceptual learning for 12 weeks.**

| **Visual perceptual learning performance** | **Total (n = 75)** | | | | | **Nunap Vision training (n = 40)** | | | | | **Nunap Vision-C training (n = 35)** | | | | |
| --- | --- | --- | --- | --- | --- | --- | --- | --- | --- | --- | --- | --- | --- | --- | --- |
|  | N | 1 week | N | 12 weeks | *p* | N | 1 week | N | 12 weeks | *p* | N | 1 week | N | 12 weeks | *p* |
| Difficulty level |  |  |  |  |  |  |  |  |  |  |  |  |  |  |  |
| Orientation | 74 | 3.61 (2.00) | 74 | 8.09 (4.01) | < .001 | 39 | 3.13 (1.90) | 39 | 9.58 (3.79) | < .001 | 35 | 4.14 (2.00) | 35 | 6.44 (3.63) | < .001 |
| Rotation | 72 | 3.17 (2.07) | 69 | 5.50 (3.52) | < .001 | 39 | 3.65 (2.41) | 37 | 6.41 (4.19) | < .001 | 33 | 2.61 (1.43) | 32 | 4.45 (2.17) | < .001 |
| Depth | 52 | 2.54 (1.11) | 42 | 3.32 (2.91) | .07 | 23 | 2.39 (1.08) | 17 | 3.84 (4.18) | .11 | 29 | 2.66 (1.13) | 25 | 2.96 (1.57) | .40 |
| Correct responses |  |  |  |  |  |  |  |  |  |  |  |  |  |  |  |
| Orientation | 74 | 57.03 (20.12) | 74 | 74.16 (16.61) | < .001 | 39 | 49.89 (14.36) | 39 | 65.85 (10.75) | < .001 | 35 | 64.98 (22.69) | 35 | 83.41 (17.20) | < .001 |
| Rotation | 72 | 55.42 (16.35) | 69 | 70.15 (15.21) | < .001 | 39 | 54.85 (17.06) | 37 | 63.85 (11.44) | < .001 | 33 | 56.11 (15.71) | 32 | 77.44 (15.93) | < .001 |
| Depth | 52 | 52.33 (15.46) | 42 | 62.22 (15.35) | .001 | 23 | 48.93 (11.01) | 17 | 57.19 (15.24) | .29 | 29 | 55.02 (17.97) | 25 | 65.64 (14.75) | < .001 |
| Overall performance |  |  |  |  |  |  |  |  |  |  |  |  |  |  |  |
| Orientation | 74 | 15.57 (11.80) | 74 | 39.28 (18.35) | < .001 | 39 | 11.78 (9.57) | 39 | 43.67 (19.40) | < .001 | 35 | 19.79 (12.72) | 35 | 34.40 (16.00) | < .001 |
| Rotation | 72 | 13.32 (11.48) | 69 | 26.52 (17.08) | < .001 | 39 | 15.41 (13.36) | 37 | 29.38 (20.76) | < .001 | 33 | 10.85 (8.29) | 32 | 23.22 (10.86) | < .001 |
| Depth | 51 | 9.71 (5.85) | 42 | 15.31 (18.03) | .03 | 23 | 8.21 (5.04) | 17 | 17.43 (26.60) | .09 | 28 | 10.95 (6.25) | 25 | 13.87 (8.87) | .07 |

The acquired training data are presented as the mean (standard deviation). Linear mixed-effects models were used to examine 12-week changes in visual perceptual learning performance within the total participants (n = 75), Nunap Vision-C training (n = 40), and Nunap Vision-C training (n = 35), respectively. The first week indicates the completion of the first block (64th trial) of the first visual perceptual learning training, and the 12 weeks indicate the completion of the last sixth block (64th trial) of the last training. Difficulty levels (1–15) were calculated by averaging those in the four quadrants, with higher numbers indicating enhanced difficulty and contrast level. Correct responses (%) were calculated within one block of 64 trials. Overall performance was calculated by multiplying the correct responses with the difficulty level divided by 15.

Abbreviations: Nunap Vision-C, Nunap Vision-Control

**Table S4. Between-group differences in visual perceptual learning performance changes.**

| **Visual perceptual learning performance** | **Nunap Vision**  **training**  **(n = 40)** | | **Nunap Vision-C**  **training**  **(n = 35)** | | *p* |
| --- | --- | --- | --- | --- | --- |
|  | N | 12-week changes | N | 12-week changes |  |
| Difficulty level |  |  |  |  |  |
| Orientation | 39 | 6.46 (4.13) | 35 | 2.29 (3.97) | < .001 |
| Rotation | 37 | 2.65 (3.82) | 32 | 1.88 (2.02) | .64 |
| Depth | 17 | 1.29 (4.11) | 25 | 0.15 (1.53) | .80 |
| Correct responses |  |  |  |  |  |
| Orientation | 39 | 15.96 (17.04) | 35 | 18.43 (25.27) | .92 |
| Rotation | 37 | 7.16 (16.27) | 32 | 21.14 (18.69) | < .001 |
| Depth | 17 | 8.24 (19.64) | 25 | 9.08 (13.96) | .70 |
| Overall performance |  |  |  |  |  |
| Orientation | 39 | 31.89 (20.91) | 35 | 14.60 (19.39) | < .001 |
| Rotation | 37 | 13.24 (19.19) | 32 | 12.42 (10.48) | .67 |
| Depth | 17 | 8.63 (26.70) | 24 | 2.55 (7.75) | .85 |

The acquired training data are presented as the mean (standard deviation). Mann–Whitney U tests were used to compare the 12-week changes in visual perceptual learning performance between the Nunap Vision and the Nunap Vision-C training groups. The changed amount during the 12-week training was calculated by subtracting the first week data from the 12-week follow-up data. The first week indicates the completion of the first block (64th trial) of the first visual perceptual learning training, and the 12 weeks indicate the completion of the last sixth block (64th trial) of the last training. Difficulty levels (1–15) were calculated by averaging those in the four quadrants, with higher numbers indicating enhanced difficulty and contrast level. Correct responses (%) were calculated within one block of 64 trials. Overall performance was calculated by multiplying the correct responses with the difficulty level divided by 15.

Abbreviations: Nunap Vision-C, Nunap Vision-Control

**Table S5. Adverse events (safety analysis set).**

| **Adverse events** | **Total** | **Nunap Vision training** | **Nunap Vision-C**  **training** | **Comment** |
| --- | --- | --- | --- | --- |
|  | (n = 79) | (n = 41) | (n = 38) |  |
| Eye disorders | 1 (1.27) | 1 (2.44) | 0 (0) |  |
| Visual acuity reduced | 1 (1.27) | 1 (2.44) | 0 (0) |  |
| Gastrointestinal disorders | 2 (2.53) | 1 (2.44) | 1 (2.63) |  |
| Nausea | 1 (1.27) | 1 (2.44) | 0 (0) | ADE |
| Vomiting | 1 (1.27) | 0 (0) | 1 (2.63) | ADE |
| Injury and procedural complications | 1 (1.27) | 1 (2.44) | 0 (0) |  |
| Concussion | 1 (1.27) | 1 (2.44) | 0 (0) |  |
| Nervous system disorders | 9 (11.4) | 4 (9.76) | 5 (13.2) |  |
| Carotid artery stenosis | 1(1.27) | 0 (0) | 1(2.63) | SAE |
| Cerebral hemorrhage | 1(1.27) | 0 (0) | 1(2.63) | SAE |
| Dizziness | 3 (3.80) | 2 (4.88) | 1 (2.63) | ADE |
| Headache | 6 (7.59) | 3 (7.32) | 3 (7.89) | ADE |
| Total | 11 (13.9) | 6 (14.6) | 5 (13.2) |  |

Data are indicated as numbers of participants (percentile).

Abbreviations: ADE, adverse device effect, Nunap Vision-C, Nunap Vision-Control; SAE, serious adverse effect.

| **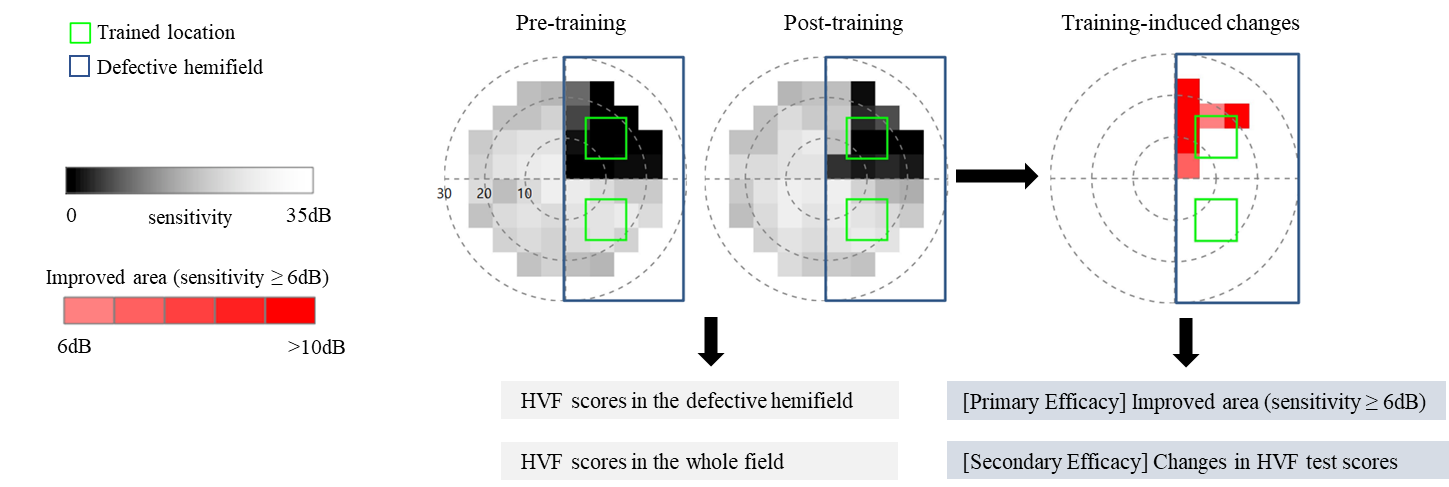** |
| --- |

**Figure S1. Study outcome measures.**

Assessment outcomes using the Humphrey visual field test in one exemplary patient with quadrantanopia, who received the Nunap Vision training. The darker visual points indicate more visual deficits with lower sensitivity, measured using Humphrey visual field tests. The green and blue boxes indicate the trained location and defective hemifield, respectively. The red color bar indicates changes in improved area (≥ 6 dB in luminance sensitivity) after the 12-week Nunap Vision training.

Abbreviations: HVF, Humphrey Visual Field test.
